# Benefits of the PRISM Shelter-Based Program for Attainment of Stable Housing and Functional Outcomes by People Experiencing Homelessness and Mental Illness: A Quantitative Analysis

**DOI:** 10.1177/07067437231162494

**Published:** 2023-03-20

**Authors:** Ghassen Soufi, Brigitte Voisard, Eric A. Latimer, Lavina Matai, Erica E. M. Moodie, Vincent Laliberté

**Affiliations:** 1 Department of Psychiatry, McGill University, Montreal, Quebec, Canada; 2 Department of Psychology, Université du Québec à Montréal (UQAM), Montreal, Quebec, Canada; 3 Psychosocial Division, Douglas Hospital Research Centre, Verdun, Quebec, Canada; 4 Department of Epidemiology, Biostatistics and Occupational Health, and Department of Equity, Ethics and Policy, McGill University, Montreal, Quebec, Canada; 5 Department of Epidemiology, Biostatistics, and Occupational Health, McGill University, Montreal, Quebec, Canada; 6 Department of Psychiatry, Jewish General Hospital, McGill University, Montreal, Quebec, Canada

**Keywords:** homeless persons, mental disorders, severe, housing, emergency shelter, urban health services, mental health services, quality of life.

## Abstract

**Objective:**

To explore the housing trajectory, personal recovery, functional level, and quality of life of clients at discharge and 1 year after completing *Projet Réaffiliation Itinérance Santé Mentale *(PRISM), a shelter-based mental health and rehabilitation program intended to provide individuals experiencing homelessness and severe mental illness with transition housing and to reconnect them with mental health and social services.

**Method:**

Housing status, psychiatric follow-up trajectory, personal recovery (Canadian Personal Recovery Outcome Measure), functional level (Multnomah Community Ability Scale), and quality of life (Lehman Quality of Life Interview) were assessed at program entry, at program discharge and 1 year later.

**Results:**

Of the 50 clients who participated in the study from May 31, 2018, to December 31, 2019, 43 completed the program. Of these, 76.7% were discharged to housing modalities and 78% were engaged with psychiatric follow-up at the program's end. Housing stability, defined as residing at the same permanent address since discharge, was achieved for 62.5% of participants at 1-year follow-up. Functional level and quality of life scores improved significantly both at discharge and at 1-year follow-up from baseline.

**Conclusions:**

Admission to PRISM helped clients secure long-term stable housing and appropriate psychiatric follow-up. Stable housing was maintained for most clients at 1-year follow-up, and they benefited from sustained functional and quality of life outcomes in long-term follow-up.

## Abrégé

**Objectif:**

Explorer la trajectoire du logement, le rétablissement personnel, le niveau fonctionnel, et la qualité de vie des clients au congé et un an après avoir terminé PRISM, un programme de santé mentale et de réhabilitation offert en refuge et visant à offrir aux personnes sans abri et souffrant de maladie mentale grave un logement transitoire et à les réengager avec des services sociaux et de santé mentale.

**Méthode:**

Le statut de logement, la trajectoire du suivi psychiatrique, le rétablissement personnel (Mesure canadienne du rétablissement personnel), le niveau fonctionnel (Échelle de capacité communautaire de Multnomah) et la qualité de vie (Échelle de la qualité de vie de Lehman) ont été évalués à l’entrée du programme, au congé du programme et un an plus tard.

**Résultats:**

Sur les 50 clients qui ont participé à l’étude du 31 mai 2018 au 31 décembre 2019, 43 ont terminé le programme. Sur ceux-ci, 76,7% étaient logés à leur congé et 78% ont été engagés dans un suivi psychiatrique à la fin du programme. La stabilité de logement, définie comme étant résider à la même adresse permanente depuis le congé, a été maintenue pour 62,5% des participants au suivi d’un an. Les scores du niveau fonctionnel et de la qualité de vie se sont améliorés significativement tant au congé qu’au suivi d’un an relativement à l'entrée au programme.

**Conclusions:**

L’admission au PRISM a aidé les clients à obtenir un logement stable à long terme et un suivi psychiatrique approprié. La stabilité de logemente a été maintenue pour la plupart des clients au suivi d’un an, et ils ont bénéficié de gains fonctionnels et de qualité de vie soutenus au suivi à long terme.

*Projet Réaffiliation Itinérance Santé Mentale* (PRISM) is a shelter-based mental health service launched in Montreal, Canada, in 2013^1,2^ as a novel approach to provide services to homeless individuals with mental illness by establishing partnerships between the city's shelters and public mental health and social service providers. There are currently three PRISMs in Montreal. Each PRISM occupies a designated area of a shelter and has a capacity of 8 to 16 individuals at a time. The aim of the program is to provide short-term residential services and on-site psychiatric care to help clients transition to permanent housing after 8 to 12 weeks and to connect them with long-term support services. On-site psychiatric and nursing care is available, and a psychoeducator/social worker as well as an intervention worker helps clients reach reintegration goals. Clients benefit from various forms of subsidized housing, a quick access to universal health coverage as well as disability income support. PRISM also refers participants to scattered-site Housing First providers. A more exhaustive report of PRISM and its services is described in Laliberté et al.^1^

The PRISM program has been the subject of two studies to date. First, a descriptive study conducted from November 18, 2013, to May 31, 2019, based on a chart review of 4 operating PRISM partnerships^1^ revealed that, of 579 clients, 63% were housed after program discharge (52% to permanent housing and 11% to temporary housing), 21% were not, and 16% were transferred to a hospital or rehabilitation centre. Furthermore, 85% had received some form of outpatient or community service support at discharge. The study also showed that 57% of clients came directly from the street or shelters, while the rest were referred by health care resources (e.g., hospitals, emergency departments, and other clinical teams). Both community and health care partners were informed about PRISM for them to refer clients most likely to benefit from the program. Second, in a qualitative study based on interviews conducted with 20 clients at the time of program admission and again at discharge,^2^ it emerged that 3 aspects of the program played a role in patient recovery: the community-based approach and its flexible structure; the possibility of taking “a break” in a secure environment that allowed focusing on one's physical and mental health; and the multiple modalities of treatment at PRISM adapted to each client, which contrasted with less desirable experiences in hospitals. Whether the benefits of PRISM are maintained in long-term follow-up after program completion remains to be investigated.

An important value of PRISM is to provide an 8- to 12-week period of stabilization in the shelter, during which the team can make a careful assessment of the needs and strengths of the clients, as well as their housing and service-related preferences. PRISM is then able to refer appropriate clients to Housing First^3–8^ or other programs. PRISM can thus act somewhat like Critical Time Intervention,^9,10^ in that it serves to help a client connect to housing and services that will enable them to function successfully in the community.

The aim of our study was 2-fold: first, to explore the housing trajectory of PRISM clients 1 year on from program completion and, second, to assess the short- and long-term benefits of program admission for client personal recovery, functional level, and quality of life (QoL). We hypothesized that participation in PRISM would lead to sustained stable housing outcomes at 1-year follow-up and both immediate and sustained improvement in quantitative questionnaire measures after program completion.

## Method

### Design

For our purposes, we undertook a prospective cohort study in which we followed a group of clients admitted to PRISM-WHM, the partnership with Welcome Hall Mission, an all-male shelter where 8 beds were reserved for the program. All clients were admitted to PRISM-WHM from a situation of homelessness, either directly from the street or a shelter or following admission to an affiliated McGill University health centre (see Table 3). Clients were met at intake, discharge, and at 1 to 2 years following discharge. At each time point, clients completed an interview with a research team member, as well as a series of questionnaires. The purpose of this approach was to explore the impact of PRISM on both short- and long-term outcomes regarding housing status, personal recovery,^11^ functional level, and QoL.

**Table 3.** Housing Status at Entry, Discharge and 1-Year Follow-up.

Housing status		Frequency	Percent
T1			
	Arrived from hospital	9	19.57
	Arrived from the street or shelter	33	71.73
	Arrived from elsewhere^a^	4	8.70
	Total	46	100
T2			
	Housing	33	76.74
	Hospitalized	5	9.30
	No housing	5	9.30
	Total	43	100
T3			
	Stable housing^b^	18	56.25
	Stable housing, but required relocation*	2	6.25
	Unstable housing**	6	18.75
	In supervised housing***	3	9.38
	No housing	3	9.38
	Total	32	100

^a^ Client staying temporarily in a transition house or with family or friends.

^b^ Defined as occupying and residing at the same permanent address since program discharge.

*Client moved at least once since PRISM discharge.

**Client had permanent housing at 1-year follow-up but experienced prolonged hospitalization or homelessness in the interim.

***Client in group home, residence, addiction resources, etc.

*Note*: Totals do not include clients who left prior to the program's end or who were unreachable.

### Recruitment

**Figure 1.**
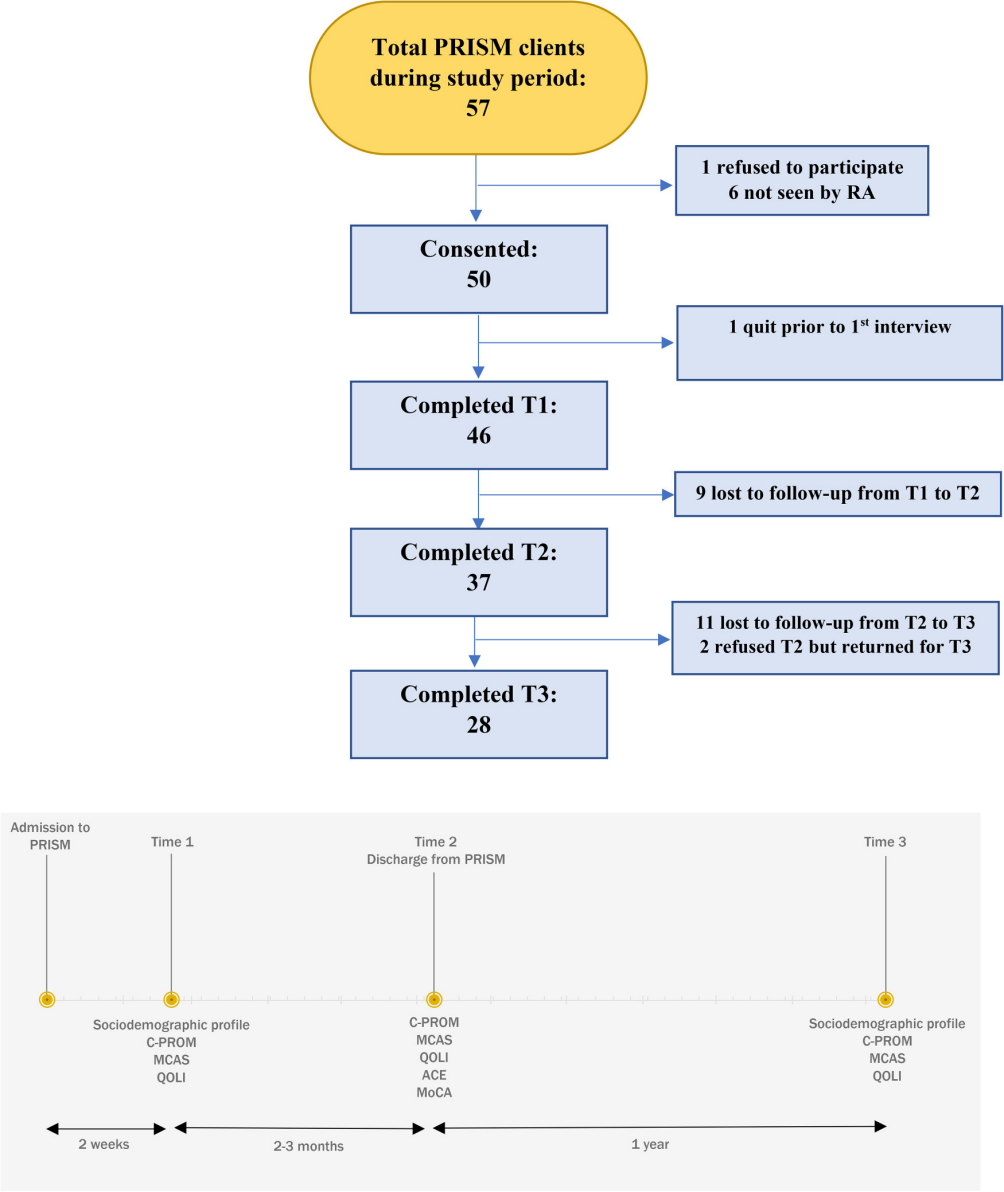
Patient recruitment and timeline.

From May 31, 2018, to December 31, 2019, every individual admitted to PRISM-WHM was eligible to participate in the study. Of these 57 individuals, 50 consented to participate in the study (see Figure 1) after meeting a member of the research team (BV) within 2 weeks of admission to the shelter. The only inclusion criterion was to be admitted to PRISM-WHM. No client was to be excluded owing to lack of fluency in French or English.

### Data Collection

Clients met at 3 different times with a member of the research team (BV) for data collection (see Figure 1): first, at program admission (T1); second, immediately upon program discharge, generally from 2 to 3 months after admission (T2); and third, 1 year after program discharge (T3). Study participants received compensation for each interview. At T3, clients were considered unreachable after several unsuccessful contact attempts, including with a second backup contact and a treating team contact, when available.

### Baseline Sociodemographic and Clinical Variables

The baseline interview included a sociodemographic component to obtain the following data: date of birth, sex, sexual orientation, income, education, substance use history, criminal justice involvement history, and housing history. Stable housing was defined as occupying and residing at the same permanent address since program discharge. Education was divided into 8 levels as per the *International Standard Classification of Education*.^12^ Substance use was divided into 3 categories based on self-reported data (see Table 1). Criminal justice involvement was categorized dichotomously based on self-reported data. The data were evaluated by 2 separate groups of authors (BV/GS and VL) who rated them independently before comparing results. Differences were discussed and resolved by consensus.

**Table 1.** Sociodemographic Data (*N*  =  50).

	Mean	*SD*
Age (years)	46.5	12.4
Education (years completed)	10.9	2.7
	Frequency	Per cent
Education level		
Lower secondary	13	35.1
Upper secondary	8	21.6
Postsecondary nontertiary	11	29.7
Short cycle tertiary	2	5.4
Bachelor's	3	8.1
Substance use		
Never problematic	8	20.5
Not recently problematic^a^	15	38.5
Recently problematic^b^	16	41.0
Lifetime justice involvement		
None^c^	9	23.1
Minor offence^d^	9	23.1
Major offence^e^	21	53.8
6-month justice involvement		
None	25	64.1
Minor offence	6	15.4
Major offence	8	20.5

^a^ Has used substances in a problematic way over a lifetime, but not in the past 6 months.

^b^ Has used substances in a problematic way in the past 6 months.

^c^ No contact with the justice system or police.

^d^ Has been fined or found guilty of misdemeanours.

^e^ Has done prison time or been found guilty of a criminal offence.

## Other Study Measures

Three other questionnaires were completed at baseline (T1), T2, and T3: the Canadian Personal Recovery Outcome Measure (C-PROM),^13,14^ the Multnomah Community Ability Scale (MCAS)^15^ to measure functional level, and the Quality of Life Inventory 20 (QOLI-20).^16,17^ Additionally, at T2, each client completed the Adverse Childhood Experiences (ACE) questionnaire^18^ and the Montreal Cognitive Assessment (MoCA).^19^ During the interview at T3, information was sought regarding service utilization, housing and hospitalization since T2. All questionnaires at all times were administered by BV, a research team member independent of the clinical team, who completed training in MCAS administration prior to patient recruitment.

### Impact of COVID-19 Pandemic on Interview Schedule

All clients were recruited and completed the intake interview (T1), the PRISM program, and the exit interview (T2) in person at the shelter prior to the introduction of public health restrictions on social contacts on March 13, 2020. Following this date, a portion of our interviews was conducted by phone (11 of 28 interviews), and compensation was paid by way of electronic bank transfer alternatively when mailbox deposits were not an option.

### Data Analysis

Client questionnaire data and sociodemographic data were analysed using both SPSS v27^20^ and R. Descriptive statistics were computed for questionnaire scores (mean and standard deviation [*SD*]) at all 3-time points and for sociodemographic variables (mean, *SD*, frequency, per cent). The MCAS and QOLI scores (see Table 2) were analysed both in their totality and in separate subcategories, as established in prior questionnaire validation studies.^10–12^ Autoregressive random effects models were used to assess changes in the questionnaire and subcategory scores across time. Weighted analyses using participants’ initial MCAS score as a predictor in the attrition model used to create the weights were also conducted to account for possible selection bias but showed no meaningful difference from the unweighted analyses. Simple linear regression analyses were subsequently completed to characterize group differences in scores between clients with stable housing at T3 and those without at T3, correcting for previous questionnaire scores at T1 and T2 as well as age and education level.

**Table 2.** Questionnaire Scores.

		T1		T2		T3	
		Mean	*SD*	Mean	*SD*	Mean	*SD*
C-PROM total		20.22	0.81	20.24	0.76	21.20	0.93
MCAS total		62.63	6.1	65.97	5.90	70.39	7.8
	Health score	20.65	2.25	21.38	2.2	21.89	1.8
	Adaptability	9.41	1.6	9.62	1.2	11.92	2.5
	Social skills	16.80	3.1	17.86	3.0	19.71	3.3
	Behaviour	15.76	2.1	17.11	1.9	16.86	2.5
QOLI-20							
	Family	3.97	1.6	3.76	1.8	4.41	1.5
	Finances	3.24	1.9	4.11	1.8	4.16	1.9
	Leisure	4.37	1.3	4.69	1.3	5.04	1.2
	Living situation	4.30	2.1	5.41	1.6	5.07	1.7
	Safety	5.34	1.1	5.64	0.9	5.73	1.2
	Social	4.36	1.5	4.62	1.4	4.80	1.5
	Q20 (Overall)	4.89	1.6	5.05	1.5	4.86	1.5
				Mean	*SD*		
ACE				4.09	2.4		
				Score ≥ *4*	Score < *4*		
				19	15		
MoCA				Mean	*SD*		
				24.16	2.5		
				Score ≥ *26*	Score *<* *26*		
				10	27		

*Note*. C-PROM = Canadian Personal Recovery Outcome Measure; MCAS = Multnomah Community Ability Scale; QOLI-20 = 20-item Quality of Life Inventory; ACE = Adverse Childhood Experience; MoCA = Montreal Cognitive Assessment; T = time; *SD* = standard deviation.

### Ethics Approval

Ethics approval for this study was obtained from the Psychosocial Research Ethics Committee of the CIUSSS West-Central Montreal Research Ethics Board.

## Results

### Baseline Characteristics

Table 1 summarizes the basic sociodemographic characteristics of the study sample. Study participants ranged in age from 19 to 65 years at T1. The mean age was 44.6 years (*SD* 12.4). Clients who completed the program spent an average of 70.7 days at PRISM-WHM. Those who were contacted at T3 were reached on average 434.5 days after program completion. A majority (55.9%) scored 4 or higher on the ACE questionnaire, *M*  =  4.09, *SD*  =  2.4, which is indicative of a significant history of childhood trauma.^21^ Only 27.0% obtained scored 26 or higher on the MoCA, *M*  =  24.16, *SD*  =  2.5, the score below which mild cognitive impairment is indicated.

### Housing and Psychiatric Follow-up Status at Program Discharge and 1-Year Follow-up

Two-thirds of all clients were discharged from the program to stable housing. Of the 43 participants who completed the program at T2, 33 (76.7%) were discharged to stable housing. Housing data were available for 32 participants at T3: at 1-year follow-up, 62.5% of these described a stable housing situation, and 56.3% were living at the same address that they had been discharged to. By the end of the program at T2, 78% of the study's 50 participants had engaged in psychiatric follow-ups of some sort.

### Functional Level, Personal Recovery, and QoL Outcomes Across Time

Our study showed an improvement in participant functional level in the time spent at PRISM-WHM, the results of which are outlined in Table 4. A significant improvement of 3.26 (95% CI, 1.35 to 5.17) was noted in MCAS mean score from T1, at program entry, to T2, at program discharge, *p*  =  0.001. There was also a significant improvement from T1 to T3 of 6.62 (95% CI, 4.21 to 9.01), *p* < 0.001, which suggested a lasting effect of these benefits 1 year after program discharge. For participants for whom we had data at T2 and T3, a significant increase in MCAS was also observed between those 2 time points, 3.35 (95% CI, 1.23 to 5.48), *p*  =  0.002.

**Table 4.** Autoregressive Random Effects Model Results.

		T1 to T2		T2 to T3		T1 to T3	
		Estimate (95% CI)	p	Estimate (95% CI)	p	Estimate (95% CI)	p
C-PROM Total		−0.03 (−1.78 to 1.72)	0.972	0.77 (−1.16 to 2.70)	0.429	0.74 (−1.21 to 2.69)	0.451
MCAS Total		3.26 (1.35 to 5.17)	0.001	3.35 (1.23 to 5.48)	0.002	6.62 (4.21 to 9.02)	<0.001
	Health score	0.73 (−0.03 to .48)	0.058	0.13 (−0.70 to 0.97)	0.209	0.86 (−0.05 to 1.76)	0.063
	Adaptability	0.21 (−0.46 to 0.88)	0.528	2.30 (1.56 to 3.03)	<0.001	2.51 (1.74 to 3.28)	<0.001
	Social skills	1.02 (0.17 to 1.87)	0.020	1.31 (0.36 to 2.25)	0.007	2.32 (1.30 to 3.34)	<0.001
	Behaviour	1.32 (0.62 to 2.01)	<0.001	−0.30 (−1.07–0.47)	0.439	1.02 (0.14 to 1.89)	0.024
QOLI-20							
	Family	−0.20 (−0.67 to 0.28)	0.410	0.58 (0.07–1.09)	0.026	0.38 (-0.19 to 0.95)	0.184
	Finances	0.65 (−0.02 to 1.33)	0.057	0.38 (−0.36 to 1.11)	0.310	1.03 (0.38 to 1.68)	0.002
	Leisure	0.17 (−0.27 to 0.61)	0.445	0.44 (−0.05 to 0.92)	0.079	0.61 (0.10 to 1.11)	0.019
	Living situation	1.01 (0.29 to 1.73)	0.006	-0.25 (-1.04–0.54)	0.530	0.76 (−0.07 to 1.60)	0.074
	Safety	0.24 (−0.12 to 0.61)	0.192	0.13 (-0.27–0.52)	0.530	0.37 (−0.09 to 0.82)	0.112
	Social	0.21 (0.39 to 0.81)	0.484	0.09 (-0.56 to 0.75)	0.777	0.30 (−0.34 to 0.95)	0.349
	Q20 (Overall)	0.15 (0.28 to 0.59)	0.486	-0.26 (-0.74 to 0.22)	0.288	−0.11 (−0.59 to 0.38)	0.665

*Note*. C-PROM = Canadian Personal Recovery Outcome Measure; MCAS = Multnomah Community Ability Scale; QOLI-20 = 20-item Quality of Life Inventory; T = time; CI = 95% confidence interval; *p* = *p*-value.

The MCAS behaviour subscore improved by 1.01 (95% CI, 0.14 to 1.89) from T1 to T2, *p* < 0.001, as did the social skills subscore by 1.02 (95% CI, 0.17 to 1.87), *p*  =  0.020, which indicated participants made progress in these domains during their stay at PRISM-WHM. Furthermore, adaptability and social skills continued to improve in the year after program discharge, as suggested by the increases in the subscores from T1 to T3 of 2.51 (95% CI, 1.74 to 3.28) for adaptability, *p* < 0.001, and 2.32 (95% CI, 1.30 to 3.34) for social skills, *p* < 0.001.

While there was no significant change in the global QOLI score from T1 to T2, a change of 0.15 (95% CI, −0.28 to 0.59), there was a significant improvement in the living situation domain of 1.01 (95% CI, 0.29 to 1.73) between T1 and T2, *p*  =  0.006, as well as the finances domain of 0.83 (95% CI, 0.17 to 1.50) between T1 and T2, *p*  =  0.015. Improvements emerged at 1-year follow-up in other domains, namely leisure by 0.61 (95% CI, 0.10 to 1.11) from T1 to T3, *p*  =  0.019, and family by 0.58 (95% CI, 0.07 to 1.09) from T2 to T3, *p*  =  0.026.

Changes observed in the C-PROM between T1 and T2 of −0.03 (95% CI, −1.78 to –1.72) and between T1 and T3 of 0.74 (95% CI, −1.21 to 2.69) were not significant.

We performed simple linear regressions to characterize group differences in questionnaire scores between clients with stable housing at T3 and those without at T3. No analysis reached statistical significance for MCAS, QOLI, or C-PROM.

## Discussion

We set out to assess the impact of program participation on housing, functional, QoL and personal recovery outcomes for PRISM clients, as well as on their capacity to reconnect with psychiatric services. This was the first study to evaluate these outcomes both at the time of program completion and at 1-year follow-up, thereby providing fresh insight into the program's effectiveness.

At T2, 43 of 50 (86%) clients completed the PRISM program and 33 of 50 (66%) or 33 of 43 (76.7%) program completers were discharged to housing. Additionally, 78% of program completers were engaged with psychiatric follow-up of varying intensity prior to program discharge. Our results are congruent with the findings of Laliberté et al.,^1^ who found, based on a chart review that 63% of program users transitioned to housing after discharge while 85% had psychiatric follow-up care immediately after program completion. Given that follow-up care in the month after psychiatric discharge can be as low as 49% for patients who need to be reassessed in that timeframe,^22^ our findings reinforce the notion that the period of stabilization that clients benefit from in PRISM translates into a better transition to stable housing and more adequate follow-up.^23^ Moreover, in qualitative interviews, PRISM clients reported that the “break” from street life was a significant boon,^2^ as it afforded time and space to take care of their physical and mental health. Furthermore, establishing an environment where clients are engaged with stable housing and psychiatric follow-up has been demonstrated to predict housing status maintenance in homeless populations suffering from mental illness.^24^

At 1-year follow-up, most clients maintained positive housing outcomes, though not necessarily the same permanent address. Of the 32 clients we reached, 18 (56.2%) had maintained stable housing and another 11 (34%) were presently housed but had experienced instability since T2 (one or more moves, long-term hospitalization, or homelessness) or were in supervised housing or group programs. Together, these numbers are quite impressive for this population, seeing how all study participants were admitted to PRISM from a precarious living situation. It has been demonstrated that homeless individuals with mental health issues do poorly without housing interventions and are at higher risk of remaining unsheltered for longer periods of time^3^ and are more likely eventually to need acute-care services.^25–27^ Our figure (66%) for permanent housing status immediately following discharge is comparable to that reported in a previous PRISM study (63%).^1^ The finding that a significant proportion of clients who emerged from the program with stable housing were able to maintain it after a year is consistent with the functional and QoL improvement over time reflected by the questionnaire scores in our study. These results echo also findings regarding Housing First interventions^28,29^ to the effect that clients who obtained stable housing also presented functional improvements.

Evidently, the benefits of the PRISM program for clients who achieve stable housing include improved functioning and QoL. This is in line with the results of other studies that showed that housing interventions improved client confidence in their living situation and led to an increase in overall well-being.^30^ In our study, program completion was associated with improved client functioning at T2. Overall MCAS scores increased from program entry to program completion, which suggests that the client overall functioning improved over that period. More specifically, client health and behaviour subscores increased significantly from T1 to T2. Lastly, client QOLI living situation subscores, too, increased significantly from T1 to T2. These findings are in line with those of a previously published qualitative paper^2^ involving the same study population, where clients found that the PRISM program afforded an opportunity to engage with a variety of services without experiencing the taxing burden of life on the street. These observed changes over the course of their stay in the program may be indicative of functional growth. The fact that the program incorporates flexible therapeutic modalities within a stable structure provides clients with a platform that enables them to access permanent housing and psychiatric follow-up more readily. These gains may be reinforced to some extent by the presence of various multidisciplinary services directly in the PRISM environment, given this population's usual poor level of engagement with such services.^31,32^ The proximity of clients to these resources is maximized while they are completing the program, thus increasing the effectiveness of the mental health care and accessory services which are known to benefit clients.^33^

Considering participants we were able to follow, functioning level and QoL outcomes were maintained at follow-up a year after program completion and even improved in the case of the MCAS total score, as well as the adaptability and social skills subscores. The finding that PRISM, an in-shelter program targeting homeless individuals with severe mental illness who engage with services after numerous unsuccessful interventions, can result in positive sustained changes in their functioning is promising and underscores the program's potential to enhance client long-term functional outcomes and improve their care trajectories. In contrast, C-PROM questionnaire scores remained stable over time. This result resembles that found in the At Home/Chez Soi study, where 2 years of recovery-oriented Housing First were not sufficient to achieve improvements on a different measure of recovery, the Recovery Assessment Scale (RAS).^34,35^ In that study, nonetheless, narrative interviews revealed meaningful differences in life trajectory during the study between the 2 groups.^36^ It may be that measures such as the RAS and C-PROM are inadequate to capture the real benefits that clients with a significant burden of life adversity experience. Other ways of measuring progress on what Deborah Padgett has called “complex recovery”^37^ may need to be found.

### Limitations

The principal limitation of our study derives from the fact that we lost participants at various follow-up points, especially from T2 to T3, and their outcomes are unknown. While the extent of the attrition made direct imputation of missing values unadvisable, as a sensitivity analysis we undertook a weighted analysis to account for the significant degree of attrition, and this did not materially alter the results. It is worth noting that considerable effort was made to reach clients at T2 and T3, including contacting backup contacts and members of care teams, and it is unlikely that more clients would have been reached had any particular study parameter been changed. Such limitations at follow-up are not unusual in long-term cohort studies of marginalized populations,^38^ and even more so when it comes to reaching individuals experiencing both homelessness and severe mental illness and who often lack stability. Ultimately, this invites caution in the interpretation of our results.

In addition, while all participants were recruited prior to the onset of the COVID-19 pandemic, new public health measures were imposed in shelters during the study. Though this forced us to make minimal changes to the study protocol, no program client reported any material obstacle to participation. Still, the measures could have had an impact on some of the services offered in the shelter and on participant follow-up.

## Conclusions

PRISM is a shelter-based mental health service intended to help clients transition from acute-care settings and homelessness to sustained stable housing and appropriate psychiatric follow-up. This study demonstrated that benefits observed at program completion and positive functional and housing outcomes are maintained for the majority of clients over long-term follow-up. In a complementary study, we plan to analyse the data in provincial administrative databases for a larger pool of PRISM clients. This will allow us to examine participant characteristics more in-depth and to track housing and service use trajectories more closely. We will also conduct qualitative interviews with clients 1 year after program completion. Vulnerable homeless people with mental illness need significant support, and PRISM creates the appropriate environment and multidisciplinary service hub where clients can start or continue on the road to recovery.
